# Differential behavioral response to predator odor in neuropathic pain in mice

**DOI:** 10.3389/fpain.2023.1283550

**Published:** 2024-01-08

**Authors:** Amalia Natsi, Mary Valkanou, Elissavet Anousi, Charalampos Labrakakis

**Affiliations:** ^1^ Department of Biological Applications and Technology, University of Ioannina, Ioannina, Greece; ^2^ Athens International Master’s Programme in Neurosciences, Department of Biology, National and Kapodistrian University of Athens, Athens, Greece; ^3^ Institute of Biosciences, University Research Center of Ioannina (URCI), Ioannina, Greece

**Keywords:** chronic pain, anxiety, spared nerve injury, predator stress, hypervigilance

## Abstract

Neuropathic pain, a type of chronic pain caused by injury or disease of the somatosensory system, affects ∼10% of the general population and is difficult to treat. It is strongly associated with mood disorder comorbidities and impairs quality of life. It was recently suggested that hypervigilance caused by chronic pain might be of advantage in some species, helping them avoid predators during injury when they are most vulnerable. Here, we sought to confirm the hypervigilance hypothesis by using two predator odor (PO) paradigms, one with transient and one with continuous odor presentation. We observed behavioral responses to PO in neuropathic and control mice in an open field setting. We find that neuropathic mice show hypervigilance to PO, confirming previous results. However, we also find increased anxiety responses to neutral odor in neuropathic mice, which manifests as maladaptive pain. This demonstrates that this maladaptive nature of pain could be an evolutionary adaptation aimed at reducing injury-induced vulnerability.

## 1 Introduction

Acute pain is of enormous value to an organism. It facilitates timely responses to potential damaging stimuli, which results in avoiding injury. Pain also protects injured body parts from further damage and promotes healing. In addition, it empowers conditional learning for new environmental dangers (1). Hence, pain awards evolutionary advantages (2), while absence of pain reduces survivability (3). By contrast, chronic pain is pain that persists beyond the healing time of the injury or disease that caused it. Because of the decline in the quality of life it causes, chronic pain is considered maladaptive. This is intensified by comorbidities, such as disturbed sleep, anxiety, and depression. Indeed, patients suffering from chronic and neuropathic pain often display mood disorders with high prevalence (4–6). Epidemiological studies show increased severity of anxiety and depression symptoms in individuals with chronic pain compared with the general population (7). Cognitive impairment is also reported by patients with chronic pains (8), including difficulty with attention and reduced working memory performance (9, 10).

Recent preclinical research is challenging the concept that chronic pain is entirely maladaptive. Instead, it suggests that nociceptive sensitization and chronic pain might maintain an adaptive function. In squid, sublethal injury generates nociceptive sensitization that induces hypervigilance, mitigating the increased predator risk (11). This hypervigilance hypothesis has also been confirmed in mammals (12). Mice with neuropathic pain show increased avoidance of predator odor (PO) compartments in a short-route/long-route food reward test. Prey have evolved sensory adaptations to recognize and avoid predators. These include POs that cause stress and induce behavioral responses, such as increased vigilance, risk assessment behaviors, avoidance, and freezing (13, 14). POs from feces, urine, and skin contain chemical components such as 2,5-dihydro-2,4,5-trimethylthiazoline (TMT), 2-phenylethylamine, and other sulfur-containing compounds that elicit fear, avoidance, and other defensive behaviors in rodents (15–17). Odors stemming from herbivores, conversely, do not cause defensive behaviors in rodents (18).

Stress and pain are tightly linked. Pain itself is a stressor, and anxiety is a comorbidity of chronic pain disorders. Stress on the other hand modulates pain, with acute stress inducing analgesia while chronic stress can cause pain hypersensitivity (19, 20). Behavioral responses to stressors in chronic pain conditions have not been well studied. Here we test how neuropathic pain influences anxiety-like responses induced by PO in mice. These experiments aim to further confirm the hypervigilance hypothesis of chronic pain.

## 2 Methods

### 2.1 Animals

All procedures were in accordance with the European Communities Council Directives 2010/63/EU and were approved by the local and institutional animal care and use committee. The experiments were carried out with 3–5-month-old male CD1 mice. Mice were divided into two surgery groups, sham operated and spared nerve injury (SNI) operated. All tests were performed between weeks 5 and 8 after surgery to ensure a chronic phenotype and the expression of anxio-depressive behaviors (21).

### 2.2 Spared nerve injury surgery

An adapted SNI, the spared tibial nerve injury, surgery was performed as previously described (22). The mice were anesthetized with 100 mg/kg Ketamine (Imalgene 1000, Merial, Germany) and 10 mg/kg Xylazine (Xylapan, Vetoquinol, France). The sural and common peroneal nerves of the left hindlimb were tightly ligated with a 6-0 silk suture (Medipac, Greece) and transected. The surgery in the sham group involved the same procedure without nerve ligation and transection.

### 2.3 Mechanical allodynia testing

Behavioral testing for neuropathic pain expression was performed a day before and at Week 5 after the surgeries. The plantar side of the left paw was stimulated with a set of eight Optihair2 von Frey filaments (Marstock, Germany) in the range 0.012–2 g. The 50% withdrawal thresholds were calculated using Dixon's up-and-down method (23).

### 2.4 Odor presentation

Open field activity was tested under two conditions: a continuous and a transient presentation of PO. In the continuous odor presentation experiments (*n* = 6 sham and *n* = 6 SNI), 200 μl of fox urine (100% concentration; Kieferle, Germany) or water (neutral control) was imbued into a cotton ball and placed within a stainless steel tea bag infuser and hang within a corner of the open field apparatus at a height of 30 cm from the floor throughout the recording. For the transient odor presentation, 250 μl of fox urine or water were sprayed using a commercial automizer spray bottle at a height of 10 cm above the open field apparatus within a 3–4 s period. Two fox urine concentrations were tested with two different groups of mice in the transient odor presentation experiments. One group was presented with a high (100%) fox urine concentration (*n* = 10 sham, *n* = 7 SNI) and another group with low (66.6% diluted with water) concentration of fox urine (*n* = 7 sham and *n* = 8 SNI).

Behavioral testing to PO was conducted at Weeks 6–8 after SNI/sham surgery. Each animal was tested with neutral odor (water) first and fox urine a week later. Fox urine presentation occurred only a single time for all animals.

### 2.5 Open field test

Mice were placed at the center of a 40 cm × 40 cm × 40 cm black plexiglass arena in a ventilated hood. The animals were habituated to the arena for 5 min. A 3-min period before odor presentation was used to normalize data. Movements of mice were video-recorded automatically by a camera mounted on top of the open field arena. Position tracking and distances traveled were analyzed with imageJ/FIJI software (National Institutes of Health) and macros developed in-house.

### 2.6 Light/dark test

The light/dark apparatus consisted of a two-chamber plexiglass box. The chambers were equally sized with dimensions 15 cm × 21 cm × 21 cm and one of the chambers was dark, while the other was exposed to light. A 6 cm × 6 cm opening connected the two chambers. The mice were placed in the dark chamber and left to explore for 5 min. The movements of mice were video-recorded and the time spent in the light compartment was manually scored. The light/dark test was conducted at Weeks 6–7 after the surgeries.

### 2.7 Open field behavior after acute restraint stress

Mice were tested in the open field two times, the first time without acute restraint and the second time, a week later, immediately after acute restraint. The open field test (OFT) lasted 10 min. For acute restraint stress, mice were immobilized for 20 min in plastic 50 ml falcon tubes in which breathing holes had been drilled. The acute restraint stress OFT experiments were conducted at Weeks 6–7 after the surgeries.

### 2.8 Statistical analysis

The behavioral data were analyzed by multivariate repeated measures ANOVA (rmMANOVA), two-way ANOVA with Sidak's multiple comparisons tests, and two sample *t*-test, using SPSS (IBM, USA) and Origin (OriginLab, USA) software. The data are expressed as mean ± SEM.

## 3 Results

**Figure 1:**
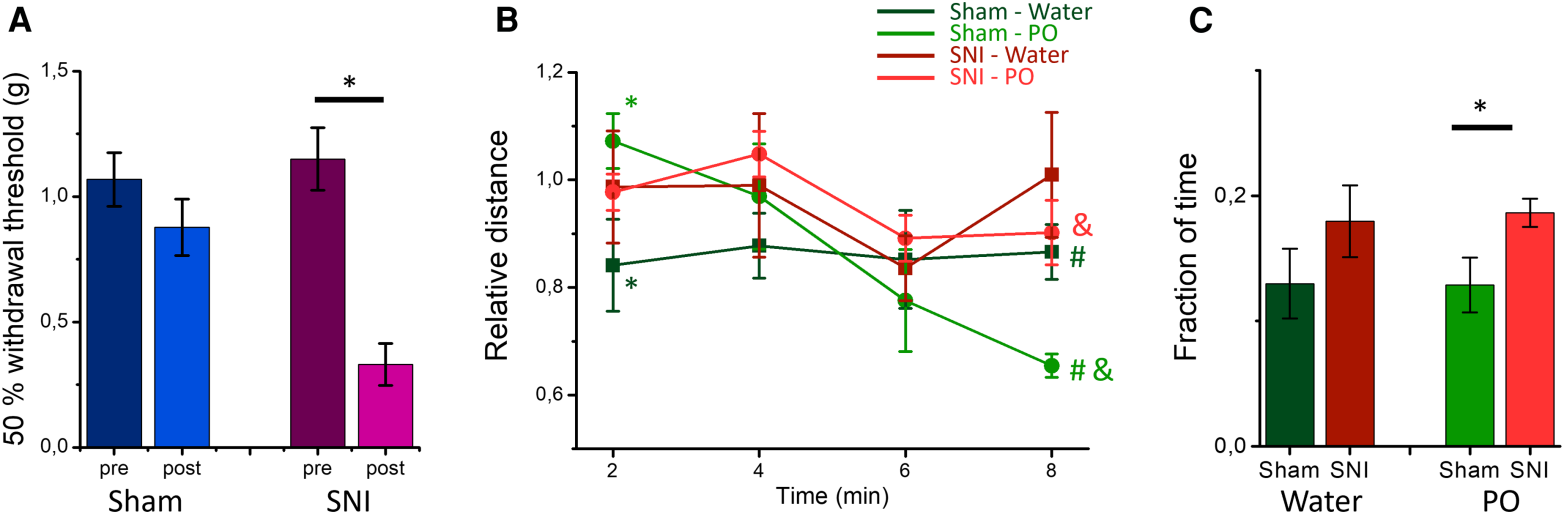
SNI and sham mice differ in their open field behavior to continuous presentation of predator odor: (**A**) sham surgery (left panel) does not produce mechanical allodynia [*F*(1, 42) = 2.298, *p* = 0.137, rmMANOVA with Sidak's multiple comparisons test] of the left hindpaw, while SNI surgery (right panel) produces mechanical hypersensitivity of the left hindpaw [*F*(1, 42) = 0.847, *p* < 0.01, rmMANOVA with Sidak's multiple comparisons test] at Week 5 after surgery. The asterisk denotes significant differences in the 50% threshold before and after surgery. (**B**) The mean time course (in 2-min bins) of the normalized distance traveled in the OFT is shown for sham (green shades) and SNI (red shades) mice presented with water (darker shades) and predator odor (PO, fox urine, lighter shades) throughout the test. * and # show significant difference between control (water) and fox urine in the sham group for the first and the last minutes, respectively, [*F*(1, 10) = 5.156, *p* = 0.047 and *F*(1, 10) = 7.710 *p* = 0.020, rmMANOVA with Sidak's multiple comparisons test] and shows significant difference in the last minutes of the PO presentation between the sham and the mice SNI [*F*(1, 10) = 14.974, *p* = 0.030, rmMANOVA with Sidak's multiple comparisons test]. (**C**) The fraction of time spent in proximity (within a 10 cm radius) of the odor source (water: dark shades, PO: light shades) for sham (green) and SNI (red) mice. * denotes significant difference between the sham and the SNI mice during PO presentation [*F*(1, 10) = 5.467, *p* = 0.041, rmMANOVA with Sidak's multiple comparisons test]. Experiments shown in (**B**) and (**C**) were conducted at weeks 6–7 after surgeries.

Control mice (sham) and mice experiencing neuropathic pain (SNI, Figure 1A) were tested for mobility as a measure of risk assessment behavior when confronted with PO. The time course of their horizontal mobility in the OFT (Figure 1B) was recorded during continuous odor presentation. Fox urine was used as a PO while water served as a control odor. The analysis with rmMANOVA showed the main effect of time [*F*(3, 8) = 5.046, *p* = 0.03] and a time and odor interaction [*F*(3, 8) = 4.382, *p* = 0.042]. Sham animals presented with water showed a stable activity throughout the 8-min period of testing. Conversely, when presented with fox urine, sham mice showed a significantly increased mobility in the first minutes of fox odor presentation, which diminished over time to a significantly lower activity compared with the control odor (Figure 1B, green shades). This signifies increased risk assessment behavior in the beginning of PO presentation. The SNI mice did not show any differences between control odor and PO but retained a relatively stable activity throughout the odor presentation. Hence, while both sham and SNI mice reacted with increased horizontal activity in the first minutes to the fox odor presentation, activity in the last minutes of the odor presentation remained high in SNI mice in comparison with the sham mice (Figure 1B), indicating a reduced habituation. We also compared the fraction of time the mice spend in the proximity of the odor source (Figure 1C), as a measure of approach behavior. A main effect of surgery was found with rmMANOVA [*F*(1, 10) = 4.991, *p* = 0.049]. Indeed, SNI mice spent a larger proportion of time in the proximity of the fox odor source than sham mice [0.19 ± 0.01 vs. 0.13 ± 0.02, respectively, *F*(1, 10) = 5.467, *p* = 0.041]. Therefore, SNI mice showing increased approach behavior also indicates increased risk assessment behavior and hypervigilance.

**Figure 2:**
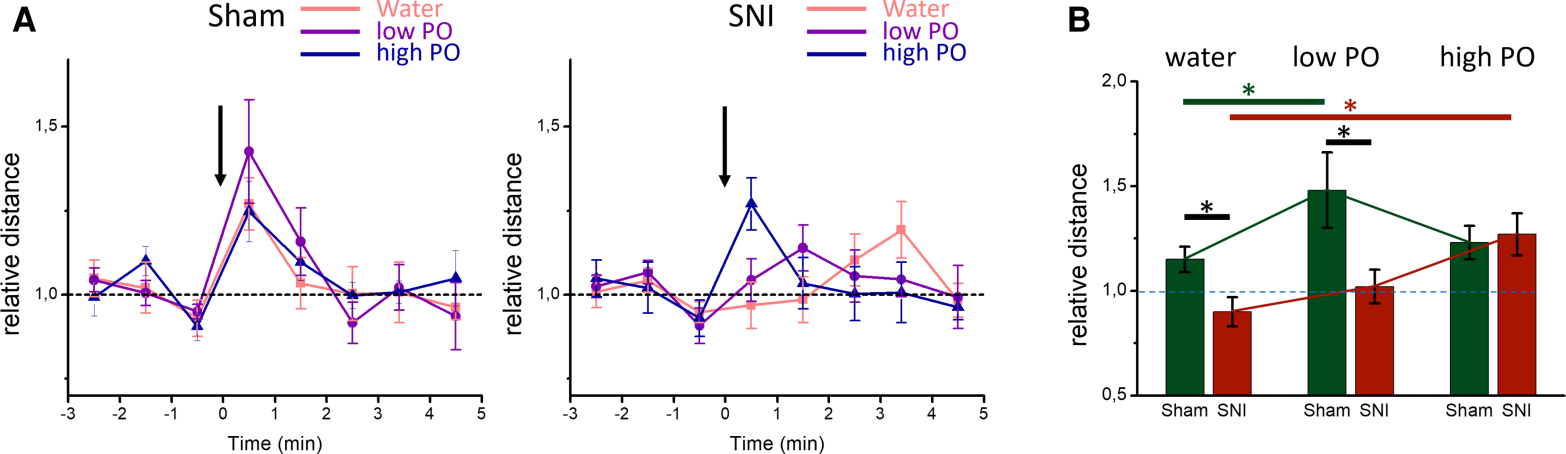
Concentration-dependent open field responses to transient presentation of PO: (**A**) mean timeline of per-minute horizontal mobility before and after transient presentation of water (pink), low (purple) and high concentration (blue) of PO for sham (left panel), and SNI (right panel) animals. (**B**) Normalized distance traveled in the OFT in the first 30 s after transient odor presentation. The sham mice (green) responded differently (*p* = 0.028, two-way ANOVA with Sidak's multiple comparison test) to low PO (66.6% fox urine) concentration compared with water, while in the SNI mice (red) the responses to high PO (100% fox urine) concentration were significantly different from that to water (*p* = 0.018). Responses to water (*p* = 0.015, two-way ANOVA with Sidak's multiple comparison test), and low concentration fox urine (*p* = 0.002) were significantly different between the sham and the SNI mice. Asterisks signify statistical differences. Experiments in A and B were conducted at Weeks 7–8 after the surgeries.

In a separate set of experiments, we assessed mouse mobility responses to short and transient PO presentation in sham and SNI mice. The horizontal mobility in the OFT was recorded before and after two different concentrations of fox urine and water control (Figure 2A). Furthermore, the analysis with rmMANOVA revealed a main effect of time [*F*(7, 50) = 8.116, *p* < 0.001] and an interaction between time and surgery [*F*(7, 50) = 3.202, *p* = 0.003], indicating differences in the behavioral responses of sham and SNI mice. The sham mice responded transiently to odor presentation with an increase in horizontal mobility that lasted 1–2 min (Figure 2A). This response was concentration-dependent, following an inverse U-shape. Indeed, analyzing the horizontal activity for the first 30 s after odor presentation, horizontal activity is significantly larger with low concentration fox urine compared with the water controls (Figure 2B). The response to a high fox urine concentration was smaller in amplitude than the response to a lower concentration, but it was not significantly different. The SNI mice responded with a delayed horizontal activity increase to the transient presentation of water and low fox urine concentration. The responses to water peaked 4 min after application, while the responses to low fox urine concentration peaked 2 min after presentation (Figure 2A). The mean mobility increase of the SNI mice to low fox urine tended to last for several minutes. When comparing responses for the first 30 s after odor presentation, the mobility increase in response to the high fox urine concentration was significantly different from that to water control in the SNI mice. In addition, the responses to water and low fox urine concentration differed significantly between SNI and the sham mice (Figure 2B). This exemplifies further that neuropathic mice respond differently to predator stress.

**Figure 3:**
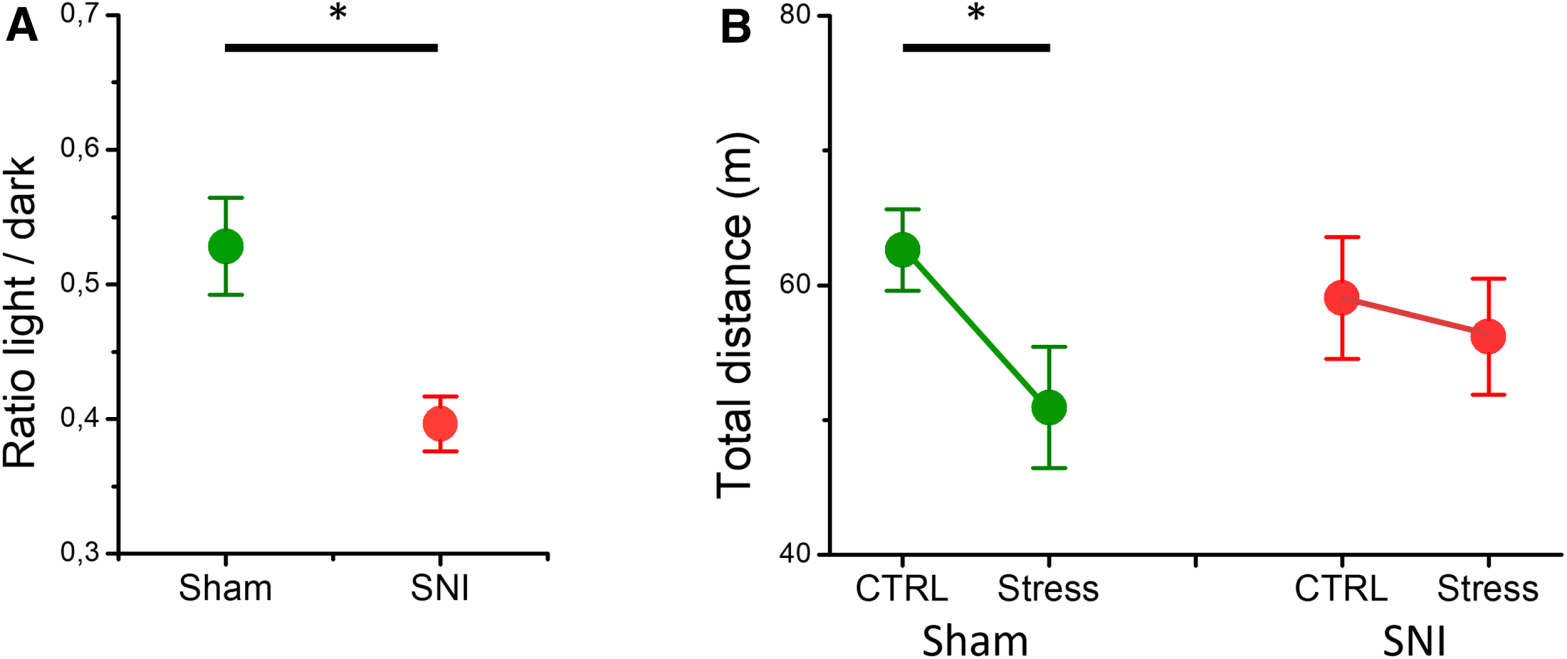
The differences in the light/dark test and acute restraint stress-induced open field behavior in the SNI and the sham mice. (**A**) The ratio of the time mice spent in the light compartment to that spent in the dark compartment is shown. The SNI mice spent less time in the light compartment (asterisk indicates a significant difference, *p* = 0.004, *t*-test). (**B**) The total distance traveled by the sham (green) and the SNI (red) mice for a 10-min period in the open field apparatus without (CTRL) and with prior restraint (Stress). The asterisk denotes significant difference in the open field behavior after acute restraint stress in the sham mice (*p* = 0.003, rmMANOVA). The experiments were conducted at Weeks 6–7 after the surgeries.

To identify if the levels of anxiety differed in the SNI and sham mice, we used the light/dark test (Figure 3A). SNI mice spent a significantly smaller ratio of time (0.39 ± 0.02, *n* = 10) in the light compartment in comparison with the sham mice (0.53 ± 0.04, *n* = 8, *p* = 0.004), indicating an increased level of anxiety in neuropathic pain. In addition, we used a different stressor to assess differences in the open field behavior of SNI and sham mice (Figure 3B). Analysis with rmMANOVA showed a main effect of acute restraint stress on total mobility [*F*(1, 26) = 7.541, *p* = 0.011]. Specifically, sham mice traveled a total distance of 62.6 ± 3.6 m in the OFT, in control conditions, but the total distance traveled significantly decreased after acute restraint stress (50.9 ± 4.3 m, *p* = 0.003, *n* = 15). Conversely, the SNI mice did not show significant differences between control and acute stress conditions in the total distance traveled (59.1 ± 3.8 m and 56.2 ± 4.6 m, respectively, *p* = 0.47, *n* = 13). Hence, neuropathic pain influences stress responses globally, independent of the nature of the stressor.

## 4 Discussion

We demonstrate that exposure to PO evokes different behavioral responses in mice with neuropathic pain, suggesting an altered processing of threat stimuli in chronic pain. Environmental stimuli that signal threat elicit defensive and anxiety-related behaviors in organisms, aiming at avoiding harm (24). These behaviors include freezing, avoidance, flight, risk assessment, and defensive attack. The particular pattern of behavior triggered dependents on several different factors like the nature of the stimulus, the distance of the stimulus source, the stimulus dynamics (for example static or moving), the specific situation, and the species. In our study, we used fox odor as a predator stimulus. Fox is a predator for mice, and mice respond to fox feces and its component 2,4,5-trimethylthiazoline with increased avoidance (25). Increased avoidance of fox urine in mice has been shown to be concentration-dependent (12). Avoidance has also been observed to fox and other predator species’ urine component 2-phenylethylamine (26). Similar results have also been reported in rats (18, 26).

We observed a complex pattern of behaviors to the presentation of fox urine in sham mice. In the beginning, these mice displayed increased mobility, a behavior reminiscent of risk assessment and vigilance, behaviors employed to detect and localize predators (13). Following this, the mice showed decreased mobility, indicating reduced exploration and a fear reaction, after recognition of the odor and predicting the presence of a predator. Decreasing risk assessment behavior with time has been shown in mice (27). Unlike other studies, we did not find increased avoidance of the fox urine area over the water controls in the sham mice. In our experiments, mice showed a comparable fraction of time approaching the odor area. This could be because of the differences in the experimental setup. We placed the source of fox urine at a distance from the floor. The distance to the threatening stimulus can be a determinant of the type of defensive response (28), with more distant threat sources triggering mainly risk assessment behavior. The use of ventilation with the OFT apparatus in our experiments could influence the dynamics of the stimulus by moving the scent away, which could influence the type of behavioral response. Indeed, threatening stimuli dynamics can dictate the freezing or fleeing response in mice, with a looming visual stimulus causing fleeing while a sweeping visual stimulus induces freezing (29). The use of a black-colored OFT apparatus might provide for a higher sense of security for the mice, thus showing more risk assessment behavior than avoidance or freezing. Another factor influencing the behavioral responses could be odor concentration; lower amounts of cat body odor, for example, do not significantly decrease contact with the odor source in rats (30). In our experiments, we did not observe freezing behavior with PO exposure. This might be owing to the mouse strain used in this study, as CD1 mice have been shown to exhibit decreased freezing during fear experiments (31).

The response of neuropathic mice to PO differed from sham controls by showing a sustained mobility pattern throughout the experiment, indicating a prolonged risk assessment behavior. This is also confirmed during the brief PO presentation in which SNI mice show a prolonged mobility response with the low fox odor concentration in comparison with the transient response of the sham mice. In addition, SNI mice show an increased approach behavior to the PO area. According to some investigators, approaching danger and risk assessment is a consequence of anxiety while the avoidance of danger and flight are the functions of fear (32). Consequently, the increased duration of risk assessment and increased approach behavior in SNI mice could be an expression of higher anxiety.

Our experiments with transient odor presentation show that there is a concentration dependence of the behavioral responses in both sham and SNI mice. However, the pattern of the dependence was different. In sham mice, the highest behavioral response was observed with low concentration of PO, while diminished responses were observed with absence of PO and high concentration odor. In the low concentration presentation, PO is a signal of higher ambiguity that probably draws higher attention and increased investigation and information-gathering (24). In the control odor presentation, mice probably recognize the absence of actual danger readily and cease risk assessment behaviors. Similarly, in the higher concentration, PO is less ambiguous and therefore rapidly recognized, while the transient nature of the signal probably leads to a rapid habituation of the behavior. In SNI mice, the absence of PO and low concentration odor showed a delay in the behavioral response, which also lasted longer, with the response to control odor peaking later than that of the low concentration PO. Only the high concentration PO showed a sharp transient response similar to that observed in sham mice. The lack of a response in the first minutes of low concentration odor could indicate a diminished response to the salient stress stimulus. A similar lack of response was also observed in the SNI mice with acute restraint stress, implying a general impact of neuropathic pain on stress responses. Such a behavioral blunting is common in depression (33). The lack of response initially and its delayed emergence could also be interpreted as a conflict between stress-induced analgesia (19) and chronic pain-induced mood comorbidities. The increased duration of the low concentration and control odor responses in the transient odor presentation experiments, together with the increased duration and approach behaviors in the continuous odor presentation experiments, is an indication of hypervigilance in the SNI mice. Hypervigilance to PO has been shown in neuropathic mice (12). They avoid the short route containing fox odor in favor of a long route toward a food reward at a higher rate than control mice. In non-mammalian species, hypervigilance also contributes to the avoidance of predators, resulting in curtailing of the increased vulnerability after injury (11). This suggests that hypervigilance during chronic pain has been evolutionarily favored by natural selection (34). Hence, for many species that are targets of attacks by predators or conspecifics, hypervigilance during chronic pain presents adaptive values. Conversely, most humans living in the modern world do not face predators or other threatening stimuli in everyday life. In the absence of predators and danger, hypervigilance equals heightened anxiety, with ambiguous and non-threatening stimuli raising the alarms. It should be noted, however, that socioeconomic stratification in human societies, which is affecting access to healthcare, safety, and nutrition, could also exacerbate pain and have an impact on vulnerability to anxio-depressive comorbidities (35, 36). In our experiments, the SNI mice showed responses to control odor presentation very similar to PO. This included prolonged mobility responses and increased approach behavior, which indicates that the SNI mice perceive controlled, non-threatening odors as an ambiguous signal, not recognizing the absence of danger, which is a manifestation of anxiety. Research on the expression of anxiety as a result of chronic pain in preclinical models has displayed conflicting and divergent results (37), with some research showing a lack of association between chronic pain and anxiety in mice (38). However, other research has demonstrated a time dependence in the expression of anxiety in chronic pain models (21, 39–41), with anxiety and other mood disorders appearing weeks after sensory changes. In our experiments, which were conducted weeks after nerve injury, the SNI mice show an increased preference for the dark compartment in the light/dark test, confirming increased anxiety with chronic pain. Anxiety disorders and depression show a strong comorbidity with chronic pain in humans (42–45). Our results reinforce the notion that chronic pain mechanisms might be a result of evolutionary forces aimed at survivability from predators, which might have lost its adaptive value in the modern world and became maladaptive. Better understanding the mechanisms linking chronic pain to mood disorders could therefore be important in improving the treatment of patients with chronic pain.

## Data Availability

The raw data supporting the conclusions of this article will be made available by the authors, without undue reservation.

## References

[1] Atlas LY. How instructions, learning, and expectations shape pain and neurobiological responses. Annu Rev Neurosci. (2023) 46:167–89. 10.1146/annurev-neuro-101822-122427 36917820 PMC11793868

[2] Nesse RM Schulkin J. An evolutionary medicine perspective on pain and its disorders. Philos Trans R Soc B Biol Sci. (2019) 374:20190288. 10.1098/rstb.2019.0288 PMC6790386 31544605

[3] Cox JJ Reimann F Nicholas AK Thornton G Roberts E Springell K An SCN9A channelopathy causes congenital inability to experience pain. Nature. (2006) 444:894–8. 10.1038/nature05413 17167479 PMC7212082

[4] Bair MJ Robinson RL Katon W Kroenke K. Depression and pain comorbidity. Arch Intern Med. (2003) 163:2433. 10.1001/archinte.163.20.2433 14609780

[5] Yalcin I Barthas F Barrot M. Emotional consequences of neuropathic pain: insight from preclinical studies. Neurosci Biobehav Rev. (2014) 47:154–64. 10.1016/j.neubiorev.2014.08.002 25148733

[6] Yalcin I Barrot M. The anxiodepressive comorbidity in chronic pain. Curr Opin Anaesthesiol. (2014) 27:520–7. 10.1097/ACO.0000000000000116 25188218

[7] Mullins PM Yong RJ Bhattacharyya N. Associations between chronic pain, anxiety, and depression among adults in the United States. Pain Pract. (2023) 23:589–94. 10.1111/papr.13220 36881021

[8] Zhang X Gao R Zhang C Chen H Wang R Zhao Q Evidence for cognitive decline in chronic pain: a systematic review and meta-analysis. Front Neurosci. (2021) 15:737874. 10.3389/fnins.2021.737874 34630023 PMC8492915

[9] Moriarty O McGuire BE Finn DP. The effect of pain on cognitive function: a review of clinical and preclinical research. Prog Neurobiol. (2011) 93:385–404. 10.1016/j.pneurobio.2011.01.002 21216272

[10] Berryman C Stanton TR Bowering JK Tabor A McFarlane A Moseley LG. Evidence for working memory deficits in chronic pain: a systematic review and meta-analysis. Pain. (2013) 154:1181–96. 10.1016/j.pain.2013.03.002 23707355

[11] Crook RJ Dickson K Hanlon RT Walters ET. Nociceptive sensitization reduces predation risk. Curr Biol. (2014) 24:1121–5. 10.1016/j.cub.2014.03.043 24814149

[12] Lister KC Bouchard SM Markova T Aternali A Denecli P Pimentel SD Chronic pain produces hypervigilance to predator odor in mice. Curr Biol. (2020) 30:R866–7. 10.1016/j.cub.2020.06.025 32750341

[13] Apfelbach R Blanchard CD Blanchard RJ Hayes RA McGregor IS. The effects of predator odors in mammalian prey species: a review of field and laboratory studies. Neurosci Biobehav Rev. (2005) 29:1123–44. 10.1016/j.neubiorev.2005.05.005 16085312

[14] McGregor IS Hargreaves GA Apfelbach R Hunt GE. Neural correlates of cat odor-induced anxiety in rats: region-specific effects of the benzodiazepine midazolam. J Neurosci. (2004) 24:4134–44. 10.1523/JNEUROSCI.0187-04.2004 15115808 PMC6729278

[15] Sievert T Laska M. Behavioral responses of CD-1 mice to six predator odor components. Chem Senses. (2016) 41:399–406. 10.1093/chemse/bjw015 26892309

[16] Rosen JB Asok A Chakraborty T. The smell of fear: innate threat of 2,5-dihydro-2,4,5-trimethylthiazoline, a single molecule component of a predator odor. Front Neurosci. (2015) 9:1–12. 10.3389/fnins.2015.00292 26379483 PMC4548190

[17] Wallace KJ Rosen JB. Predator odor as an unconditioned fear stimulus in rats: elicitation of freezing by trimethylthiazoline, a component of fox feces. Behav Neurosci. (2000) 114:912–22. 10.1037/0735-7044.114.5.912 11085605

[18] Fendt M. Exposure to urine of canids and felids, but not of herbivores, induces defensive behavior in laboratory rats. J Chem Ecol. (2006) 32:2617–27. 10.1007/s10886-006-9186-9 17131189

[19] Butler RK Finn DP. Stress-induced analgesia. Prog Neurobiol. (2009) 88:184–202. 10.1016/j.pneurobio.2009.04.003 19393288

[20] Rivat C Becker C Blugeot A Zeau B Mauborgne A Pohl M Chronic stress induces transient spinal neuroinflammation, triggering sensory hypersensitivity and long-lasting anxiety-induced hyperalgesia. Pain. (2010) 150:358–68. 10.1016/j.pain.2010.05.031 20573451

[21] Yalcin I Bohren Y Waltisperger E Sage-Ciocca D Yin JC Freund-Mercier MJ A time-dependent history of mood disorders in a murine model of neuropathic pain. Biol Psychiatry. (2011) 70:946–53. 10.1016/j.biopsych.2011.07.017 21890110

[22] Shields SD Eckert WA Basbaum AI. Spared nerve injury model of neuropathic pain in the mouse: a behavioral and anatomic analysis. J Pain. (2003) 4:465–70. 10.1067/S1526-5900(03)00781-8 14622667

[23] Dixon WJ. Efficient analysis of experimental observations. Annu Rev Pharmacol Toxicol. (1980) 20:441–62. 10.1146/annurev.pa.20.040180.002301 7387124

[24] Blanchard DC Blanchard RJ. Chapter 2.4: defensive behaviors, fear, and anxiety. In: Blanchard RJ Blanchard DC Griebel G Nutt D, editors. Handbook of Behavioral Neuroscience. Amsterdam, Netherlands: Elsevier (2008). p. 63–79. 10.1016/S1569-7339(07)00005-7

[25] Buron G Hacquemand R Pourie G Lucarz A Jacquot L Brand G. Comparative behavioral effects between synthetic 2,4,5-trimethylthiazoline (TMT) and the odor of natural fox (*Vulpes vulpes*) feces in mice. Behav Neurosci. (2007) 121:1063–72. 10.1037/0735-7044.121.5.1063 17907837

[26] Ferrero DM Lemon JK Fluegge D Pashkovski SL Korzan WJ Datta SR Detection and avoidance of a carnivore odor by prey. Proc Natl Acad Sci U S A. (2011) 108:11235–40. 10.1073/pnas.1103317108 21690383 PMC3131382

[27] Papes F Logan DW Stowers L. The vomeronasal organ mediates interspecies defensive behaviors through detection of protein pheromone homologs. Cell. (2010) 141:692–703. 10.1016/j.cell.2010.03.037 20478258 PMC2873972

[28] Blanchard DC Griebel G Blanchard RJ. Mouse defensive behaviors: pharmacological and behavioral assays for anxiety and panic. Neurosci Biobehav Rev. (2001) 25:205–18. 10.1016/S0149-7634(01)00009-4 11378177

[29] De Franceschi G Vivattanasarn T Saleem AB Solomon SG. Vision guides selection of freeze or flight defense strategies in mice. Curr Biol. (2016) 26:2150–4. 10.1016/j.cub.2016.06.006 27498569

[30] Takahashi LK Nakashima BR Hong H Watanabe K. The smell of danger: a behavioral and neural analysis of predator odor-induced fear. Neurosci Biobehav Rev. (2005) 29:1157–67. 10.1016/j.neubiorev.2005.04.008 16095694

[31] Adams B Fitch T Chaney S Gerlai R. Altered performance characteristics in cognitive tasks: comparison of the Albino ICR and CD1 mouse strains. Behav Brain Res. (2002) 133:351–61. 10.1016/S0166-4328(02)00020-7 12110469

[32] McNaughton N Corr PJ. A two-dimensional neuropsychology of defense: fear/anxiety and defensive distance. Neurosci Biobehav Rev. (2004) 28:285–305. 10.1016/j.neubiorev.2004.03.005 15225972

[33] Christensen MC Ren H Fagiolini A. Emotional blunting in patients with depression. Part I: clinical characteristics. Ann Gen Psychiatry. (2022) 21:10. 10.1186/s12991-022-00387-1 35379283 PMC8981644

[34] Walters ET. Adaptive mechanisms driving maladaptive pain: how chronic ongoing activity in primary nociceptors can enhance evolutionary fitness after severe injury. Philos Trans R Soc B Biol Sci. (2019) 374:20190277. 10.1098/rstb.2019.0277 PMC6790390 31544606

[35] Braveman PA Cubbin C Egerter S Williams DR Pamuk E. Socioeconomic disparities in health in the United States: what the patterns tell US. Am J Public Health. (2010) 100:S186–96. 10.2105/AJPH.2009.166082 20147693 PMC2837459

[36] Rios R Zautra AJ. Socioeconomic disparities in pain: the role of economic hardship and daily financial worry. Heal Psychol. (2011) 30:58–66. 10.1037/a0022025 PMC3077089 21299295

[37] Liu MG Chen J. Preclinical research on pain comorbidity with affective disorders and cognitive deficits: challenges and perspectives. Prog Neurobiol. (2014) 116:13–32. 10.1016/j.pneurobio.2014.01.003 24444673

[38] Pitzer C La C Treede PR. Inflammatory and neuropathic pain conditions do not primarily evoke anxiety-like behaviours in C57BL/6 mice. Eur J Pain. (2019) 23:285–306. 10.1002/ejp.1303 30098102

[39] Sieberg CB Taras C Gomaa A Nickerson C Wong C Ward C Neuropathic pain drives anxiety behavior in mice, results consistent with anxiety levels in diabetic neuropathy patients. Pain Reports. (2018) 3:1–11. 10.1097/PR9.0000000000000651 PMC5999418 29922743

[40] Dimitrov EL Tsuda MC Cameron HA Usdin TB Cameron HA Dimitrov EL. Anxiety- and depression-like behavior and impaired neurogenesis evoked by peripheral neuropathy persist following resolution of prolonged tactile hypersensitivity. J Neurosci. (2014) 34:12304–12. 10.1523/JNEUROSCI.0312-14.2014 25209272 PMC4160769

[41] Suzuki T Amata M Sakaue G Nishimura S Inoue T Shibata M Experimental neuropathy in mice is associated with delayed behavioral changes related to anxiety and depression. Anesth Analg. (2007) 104:1570–7. 10.1213/01.ane.0000261514.19946.66 17513660

[42] Radat F Margot-Duclot A Attal N. Psychiatric co-morbidities in patients with chronic peripheral neuropathic pain: a multicentre cohort study. Eur J Pain (United Kingdom). (2013) 17:1547–57. 10.1002/j.1532-2149.2013.00334.x 23720357

[43] Gureje O Von Korff M Kola L Demyttenaere K He Y Posada-Villa J The relation between multiple pains and mental disorders: results from the world mental health surveys. Pain. (2008) 135:82–91. 10.1016/j.pain.2007.05.005 17570586

[44] Attal N Lanteri-Minet M Laurent B Fermanian J Bouhassira D. The specific disease burden of neuropathic pain: results of a French nationwide survey. Pain. (2011) 152:2836–43. 10.1016/j.pain.2011.09.014 22019149

[45] Knaster P Estlander AM Karlsson H Kaprio J Kalso E. Temperament traits and chronic pain: the association of harm avoidance and pain-related anxiety. PLoS One. (2012) 7:e45672. 10.1371/journal.pone.0045672 23133510 PMC3485083

